# Proposing a hybrid metaheuristic optimization algorithm and machine learning model for energy use forecast in non-residential buildings

**DOI:** 10.1038/s41598-022-04923-7

**Published:** 2022-01-20

**Authors:** Ngoc-Tri Ngo, Thi Thu Ha Truong, Ngoc-Son Truong, Anh-Duc Pham, Nhat-To Huynh, Tuan Minh Pham, Vu Hong Son Pham

**Affiliations:** 1 Faculty of Project Management, The University of Danang-University of Science and Technology, 54 Nguyen Luong Bang, Da Nang, Vietnam; 2 Department of Civil Engineering, The University of Danang-University of Technology and Education, 48 Cao Thang Street, Da Nang, Vietnam; 3 Information Technology Faculty, The University of Danang-University of Science and Technology, 54 Nguyen Luong Bang, Da Nang, Vietnam; 4 Construction Engineering & Management Department, Ho Chi Minh City University of Technology, Vietnam National University-Ho Chi Minh City (VNUHCM), 268 Ly Thuong Kiet, Ho Chi Minh City, Vietnam

**Keywords:** Civil engineering, Electrical and electronic engineering

## Abstract

The building sector is the largest energy consumer accounting for 40% of global energy usage. An energy forecast model supports decision-makers to manage electric utility management. Identifying optimal values of hyperparameters of prediction models is challenging. Therefore, this study develops a novel time-series Wolf-Inspired Optimized Support Vector Regression (WIO-SVR) model to predict 48-step-ahead energy consumption in buildings. The proposed model integrates the support vector regression (SVR) and the grey wolf optimizer (GWO) in which the SVR model serves as a prediction engine while the GWO is used to optimize the hyperparameters of the SVR model. The 30-min energy data from various buildings in Vietnam were adopted to validate model performance. Buildings include one commercial building, one hospital building, three authority buildings, three university buildings, and four office buildings. The dataset is divided into the learning data and the test data. The performance of the WIO-SVR was superior to baseline models including the SVR, random forests (RF), M5P, and decision tree learner (REPTree). The WIO-SVR model obtained the highest value of correlation coefficient (R) with 0.90. The average root-mean-square error (RMSE) of the WIO-SVR was 2.02 kWh which was more accurate than those of the SVR model with 10.95 kWh, the RF model with 16.27 kWh, the M5P model with 17.73 kWh, and the REPTree model with 26.44 kWh. The proposed model improved 442.0–1207.9% of the predictive accuracy in RMSE. The reliable WIO-SVR model provides building managers with useful references in efficient energy management.

## Introduction

Electricity demand forecasting is an essential task since it supports making important decisions in power system planning and operation. Overestimation of energy usage results in unnecessary establishments and spinning reserves, inefficient load distributions, and an increase in the operation cost. In contrast, underestimation of consumption leads to insufficient reserves and high costs in the peaking unit, which limits economic and industrial development^1^. The building sector has considered the largest consumer of energy that accounts for nearly 40% of global energy usage and 33% of greenhouse gas emissions^2^. This mainly results from the population explosion worldwide and the industrial growth in past decades. An accurate and robust forecast model for building energy consumption, thus, is of importance for energy efficiency management.

Three main categories for modeling and forecasting building energy consumption are physics-based, conventional and machine learning (ML) models. Physics-based models are known as “white box” that use physics equations and thermodynamic rules to describe the building energy behavior. The accuracy of such models is low. Conventional models, which apply statistical principles, are reasonably accurate for near-linear time-series data. They include regressions-based models and auto-regressive models.

ML models have become a mainstream of modeling building energy. They are a branch of artificial intelligence (AI) and are referred to as a “black box” that can learn from historical energy use patterns to predict energy consumption^3^. ML provides humans with a mechanism to analyze huge amounts of data^4^. With the capacity in nonlinear approximation, ML models obtain better predictive performance than statistical models do^5,6^. Two sub-divisions of the ML models are single models and combined models. Representatives of individual models are artificial neural networks (ANNs)^7^, support vector machines (SVMs)^8^, random forest (RF)^9^, self-adaptive probabilistic neural network^10^, and self-adaptive extreme learning machine^11^.

Combined models include ensemble and improved models. The ensemble model builds a framework that takes advantage of the strengths and weaknesses of single machine learners to obtain better prediction performance compared to a single model^12^. Voting, bagging, RF, decision tree (DT), gradient boosting are popular ensemble learners^13^. Chen et al. used fuzzy C-means clustering and nonlinear regression to develop a day-ahead prediction model of hourly energy consumption in the building sector^14^.

Studies from literature indicated that the accuracy of ML models depends on their hyperparameters’ setting. This disadvantage could be overcome by improved models in which an optimization algorithm is employed to appropriately tune hyperparameters of the single ML model^6^. Particle swarm optimization (PSO) and genetic algorithm (GA) are often used to improve the performance of ML models. In recent years, nature-inspired algorithms that mimic the behavior of swarms in nature have been widely adopted to solve optimization problems. Developed by Mirjalili et al.^15^, a grey wolf optimizer (GWO) is a swarm-based metaheuristic algorithm that stimulates the leadership hierarchy and hunting behavior of grey wolves. The GWO algorithm has been proven its capacity in improving the performance of machine leaners in various fields^16^. For example, a fractional time delayed grey model optimized by the GWO was used to predict the natural gas and coal consumption^16^.

Identifying optimal values of hyperparameters of ML models is challenging. Therefore, this study integrates the GWO in the prediction model to optimize hyperparameters of the SVR model, which can improve the predictive performance in the prediction. Particularly, this study develops a hybrid model of wolf-inspired optimized support vector regression (WIO-SVR) for energy demand forecasting in non-residential buildings. The proposed model integrates the SVR and GWO in which the SVR model serves as a prediction engine while GWO is used to optimize the hyperparameters of the SVR model.

There are several feasible alternatives for energy consumption forecast in buildings. They may short-term prediction or long-term prediction. The study focuses on the short-term energy consumption prediction in non-residential buildings. Particularly, 1-day-ahead energy consumption prediction in 30-min intervals was adopted in this study. This means that the 48-step-ahead prediction is produced by the proposed model. Energy consumption data were collected in 30-min intervals in Danang city in Vietnam. The data were used to validate the predictive performance of the WIO-SVR. The performance of the proposed model is compared with those of benchmark models. This study contributes to (i) the state of the knowledge by examining the performance of WIO-SVR model in predicting time-series energy data; and (ii) the state of practice by proposing an effective tool to help building owners and facility managers in understanding building energy performance for enhancing the energy efficiency in buildings.

The rest of this study is organized as follows. “Previous works” reviews literature relating to energy forecast. “Methods” elucidates methodologies including the support vector regression (SVR), GWO, the hybrid of GWO and SVR, and criteria for predictive accuracy evaluation. The collection of data and analytical results are presented in “Results”. Finally, “Conclusions” summarizes conclusions and recommendations for future research.

## Previous works

The challenges of energy consumption forecasting are data’s nature (i.e., non-linear, non-stationary, and multi-seasonality) and dependence on influential factors like weather conditions, time, occupancy, etc.^17^. Physics-based and conventional models, thus, are inefficient and ineffective in modeling large building profiles. Data-driven approaches like ML models have been widely used to solve high-dimensional energy data^5,18^. ML and AI have been applied in various fields^19^ such as fish species identification^20^, rainfall prediction^21^, and energy reduction and greenhouse gas emissions mitigation^22^.

An accurate energy demand forecast model supports the decision-makers to carry out short-term management of the electric utility. In the study of Zhu et al.^5^, popular ML techniques such as LASSO regression, RF, CART, SVR were utilized to predict daily electrical load in non-residential buildings. Residuals of prediction models were then analyzed by a statistical quality control theory to monitor and identify abnormal load patterns. Biswas et al.^23^ compared various neural network-based models in predicting residential building energy usage. The inputs include the number of days, outdoor temperature, and solar radiation while the outputs consist of house and heat pump energy consumption.

Ensemble models, such as RF, DT, gradient boosting have emerged as a promising approach for modeling building energy data. Lu et al. developed a tree-based ML model-extreme gradient boosting (so-called XGBoost) to predict daily electrical load from the City of Bloomington Intake Tower in Indiana, USA^24^. This tower plays an important role in residents’ lives since its function in collecting water from reservoirs and transporting it to hydroelectric power plants or water treatment plants. The complete ensemble empirical mode decomposition with the adaptive noise (CEEMDAN) method was applied for decomposing energy patterns. Analytical results indicated that the CEEMDAN-XGBoost model outperformed benchmark models in all measures. Ma^25^ proposed a hybrid deep meta-ensemble networks to forecast hourly electric load data of 20 zones at a US utility. Experimental results presented that the forecast ability of the proposed model is superior to that of multi-layer perceptron, RF, and gradient boosting regression trees.

Pham et al.^13^ investigated the effectiveness of RF, M5P, and Random Tree (RT) in predicting short-term energy consumption in multiple buildings. Five datasets in hourly intervals collected in one year were utilized to validate the predictive ability of ML prediction models. Experimental results showed that the RF model was superior to the M5P and RT models in 1-step-ahead energy usage prediction. Pan et al.^12^ proposed a new novel ensemble model namely categorical boosting (CatBoost) to estimate multi-dimensional energy consumption data. Findings revealed a better forecast performance of the CatBoost than the RF and gradient boosting decision tree.

The predictive accuracy of the machine learner depends on tuning its parameters. The selection of such parameters is a real-world optimization problem. Metaheuristic algorithms have been widely adopted to optimize the parameters of the ML models. For example, Somu et al.^17^ introduced a combined model for short, mid, and long-term energy consumption forecasting at an academic building. The long short-term memory networks were optimized by the improved sine cosine optimization algorithm. Comparative results indicated the outperformance of the proposed forecast model to single models such as the autoregressive integrated moving average (ARIMA), Deep Belief Network regression, SVR.

Ma et al.^26^ established a modified convolutional neural network (CNN) for the interval forecasting of electricity demand. In the proposed model, the whale optimization algorithm (WOA) was employed to optimize parameters of the CNN and comprehensively evaluated calibration and sharpness of the probabilistic forecasting performance. For daily electric load forecasting of an office building, Ying et al.^27^ proposed a backpropagation (BP) neural network optimized by the GWO. Moreover, the fuzzy C-means (FCM) clustering algorithm was utilized to cluster historical energy profiles. Empirical results indicated that the FCM-GWO-BP model significantly improved the prediction accuracy of the pure BP model and the GWO-BP model without clustering.

Various studies have adopted a composite prediction model of a neural network and a metaheuristic algorithm. However, the neural networks have some limitations such as local minimum traps, slow convergence speed, and difficulty in determining the size of the hidden layer and learning rate^28^. Thus, the least square SVR is a promising alternative. Besides that, the literature showed that few studies focused on the combination of the GWO and SVR for non-residential building energy usage forecasting. In this study, the authors proposed a time-series WIO-SVR model to predict energy consumption in non-residential buildings. This proposed model takes advantage of a metaheuristic optimization algorithm (i.e., the GWO) and a machine learning model (i.e., SVR). The GWO is used to optimize the hyperparameters of the SVR model during the learning process. Thus, the integration of GWO and SVR in the proposed hybrid model can enhance the predictive performance in predicting energy use consumption in non-residential buildings. The contributions of this study are: (1) a time-series wolf-inspired optimized support vector regression (WIO-SVR) model for energy consumption prediction in non-residential buildings; (2) investigate the capacity of the WIO-SVR in electricity consumption forecasting in non-residential buildings.

## Methods

### Support vector regression for time-series data analytics

**Figure 1:**
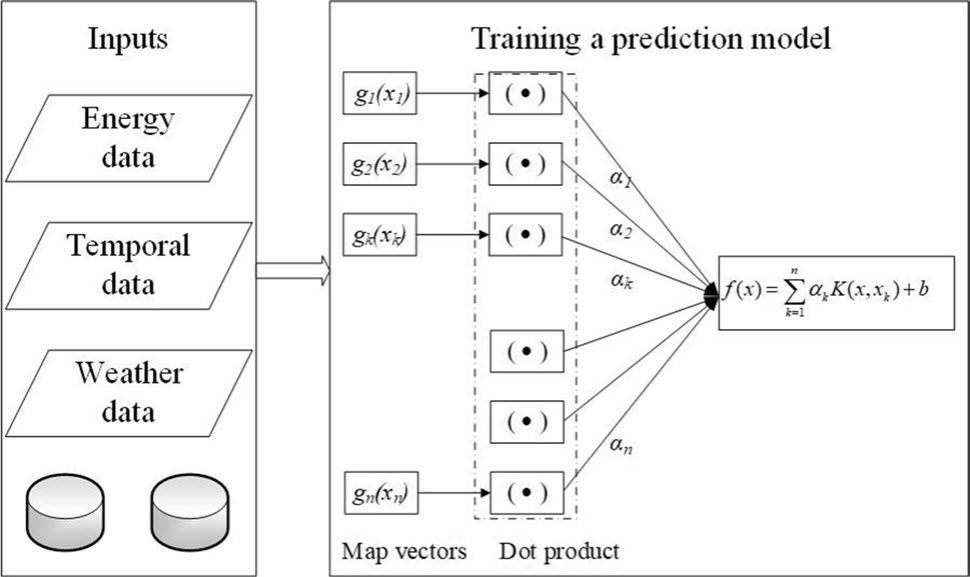
Training support vector regression model using energy data.

The support vector regression (SVR)^8^ is a supervised learning model belong to machine learning, that is used for regression problems. It has been used for capturing the non-linear relationship between the predictors and dependent variables. Figure 1 demonstrates a framework of the SVR model. Inputs for training the SVR model in this study are historical energy data, temporal data, and weather data. Future energy consumption in buildings is a prediction output of the SVR model.

The SVR model uses a kernel function to maps predictors to high-dimension feature space. A least-squares cost function is applied to train an SVR model to yield linear equations in a dual space that reduces computing time. Particularly, SVR models are taught by solving Eq. (1).

1 $$\mathop {\min }\limits_{\omega ,b,e} J(\omega ,b,e) = \frac{1}{2}\left\| \omega \right\|^{2} + \frac{1}{2}C\sum\limits_{k = 1}^{n} {e_{k}^{2} } ; \, \,\,{\text{subject}}\,{\text{ to}}\,y_{k} = \left\langle {\omega ,\varphi (x_{k} )} \right\rangle + b + e_{k} ,\quad k = 1, \ldots n,$$

where *J*(*ω*,*b*,*e*) is an objective function; *ω* is a linear approximator’s parameter; *e*_*k*_ is errors; $C \ge 0$ is a regularization parameter; *x*_*k*_ is predictors (i.e., historical energy data, temporal data, and weather data); *y*_*k*_ is dependent variables (i.e., energy consumption in buildings); *b* is bias; and *n* is the dataset size.

Lagrange multipliers (*α*_*k*_) is utilized for dealing with this problem that results in Eq. (2). A kernel function is described in Eq. (3) in which the Gaussian radial basis functions (RBF) kernel as Eq. (4) was used. The RBF kernel was adopted as the kernel function because it has low mathematical complexity and effectively solves a highly nonlinear problem^29^.

2 $$f(x) = \sum\limits_{k = 1}^{n} {\alpha_{k} K(x,x_{k} )} + b,$$

3 $$K(x,x_{k} ) = \sum\limits_{k = 1}^{n} {g_{k} (x)g_{k} (x_{k} )} ,$$

4 $$K(x,x_{k} ) = \exp ( - \left\| {x - x_{k} } \right\|^{2} /2\sigma^{2} ),$$

where *α*_*k*_ are Lagrange multipliers; *K(x,x*_*k*_*)* is the kernel function; *σ* is the RBF width.

Performance of SVR models is affected by the value settings of its hyperparameters that consists of the RBF width (*σ*) and the regularization parameter (*C*). In this study, the optimal settings of these two hyperparameters were considered comprehensively. Particularly, a recently developed metaheuristic grey wolf optimization (GWO) algorithm^15^ was integrated to optimize the performance of the proposed WIO-SVR model. The mathematical theory of GWO was presented in the next section.

### *Wolf-inspired optimization for* enhancing machine learning model

**Figure 2:**
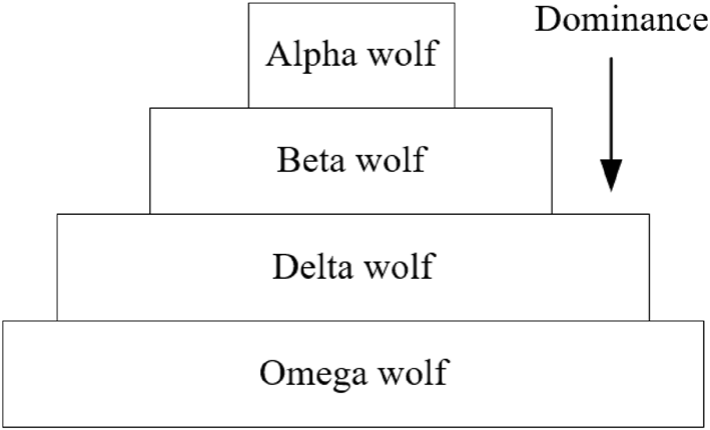
Dominant structure in a grey wolf swarm.

The GWO is a metaheuristic optimization algorithm^15^ that inspires the natural behaviors of grey wolves. The GWO follows the power hierarchy and hunting activities of wolves. Particularly, a swarm of grey wolfs is split hierarchically into four subswarms of alphas (*α*), beta (*β*), delta (*δ*), and omega (*ω*) in which power and responsibility of each group are different. Figure 2 shows the dominant structure in a grey wolf swarm.

- Alpha is a leader.
- Beta is subordinate wolves that consult the alpha and manage the pack.
- Delta is wolves that need to report to the alpha and betas.
- Omega plays as a scapegoat and reports to the alpha, beta, and delta.

**Figure 3:**
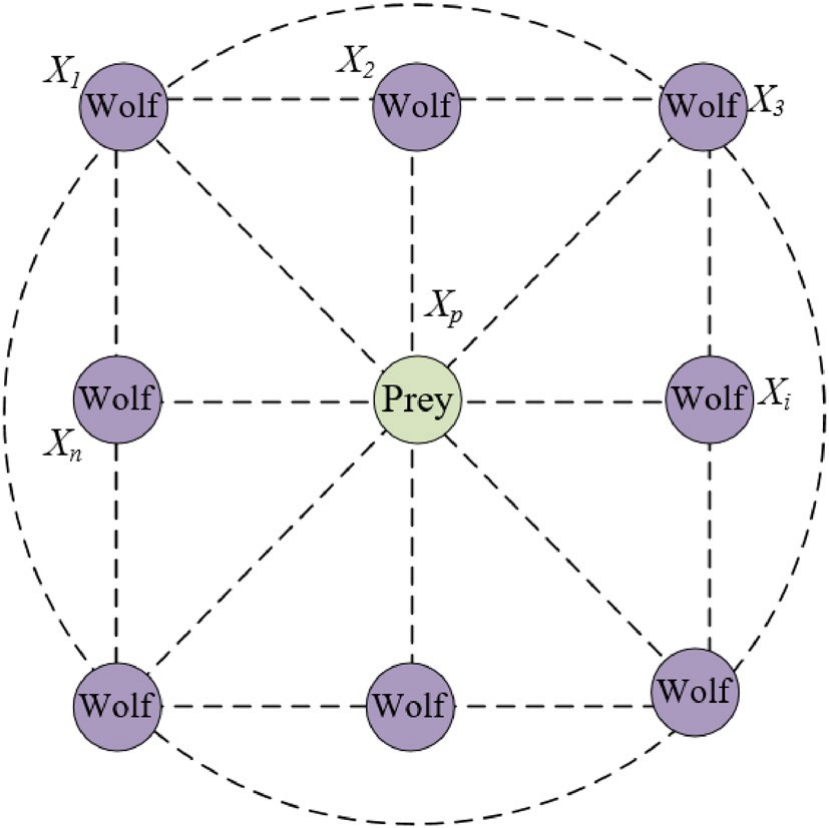
Illustration of positions of grey wolves and prey.

For modeling the social power of wolves, the *α* is considered as the fittest solution; *β* and *δ* are considered as the 2-nd and 3-rd third best solutions, respectively^15^. The optimization by the GWO includes searching, encircling, and attacking prey. Figure 3 illustrates of positions of grey wolves and prey. The hunting process is led by *α*, *β*, and *δ*. Encircling prey was performed by updating the wolf position by using by Eqs. (5) and (6). Three of them were used to predict the location of the grey while the location of omegas was updated randomly surrounding the three best wolves as shown in Eqs. (7)–(10) ^15^.

5 $$\vec{X}(t + 1) = \vec{X}_{p} (t) - \vec{A} \cdot \left| {\vec{C} \cdot \vec{X}_{p} (t) - \vec{X}(t)} \right|,$$

6 $$\vec{A} = 2\vec{a} \cdot \vec{r}_{1} - \vec{a}\,{\text{and}}\,\vec{C} = 2 \cdot \vec{r}_{2} ,$$

7 $$\vec{X}_{1} = \vec{X}_{\alpha } - \vec{A}_{1} \cdot \overrightarrow {D}_{\alpha } ,\,\overrightarrow {D}_{\alpha } = \left| {\vec{C}_{1} \cdot \vec{X}_{\alpha } - \vec{X}} \right|,$$

8 $$\vec{X}_{2} = \vec{X}_{\beta } - \vec{A}_{2} \cdot \overrightarrow {D}_{\beta } ,\,\overrightarrow {D}_{\beta } = \left| {\vec{C}_{2} \cdot \vec{X}_{\beta } - \vec{X}} \right|,$$

9 $$\vec{X}_{3} = \vec{X}_{\delta } - \vec{A}_{3} \cdot \overrightarrow {D}_{\delta } ,\,\overrightarrow {D}_{\delta } = \left| {\vec{C}_{3} \cdot \vec{X}_{\delta } - \vec{X}} \right|,$$

10 $$\vec{X}(t + 1) = \frac{{\vec{X}_{1} + \vec{X}_{2} + \vec{X}_{3} }}{3},$$

where $\vec{X}(t + 1)$ is a location vector of wolves at iteration (*t* + 1); $\vec{X}_{p} (t)$ is a location vector of prey at iteration *t*; $\vec{A}$ and $\vec{C}$ are coefficient vectors; $\vec{a}$ are reduced from 2 to 0 through iterations; $\vec{r}_{1}$ and $\vec{r}_{2}$ are random vectors of [0, 1].

**Figure 4:**
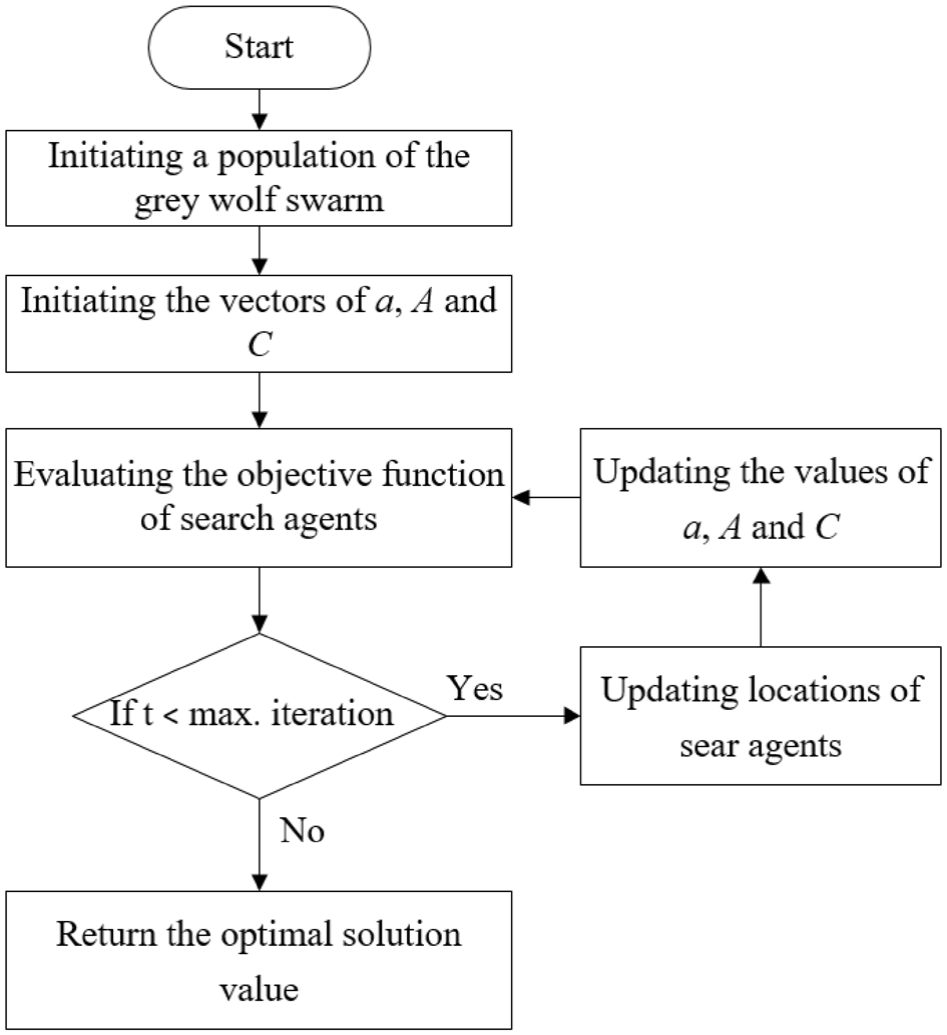
The flowchart of the grey wolf optimization algorithm.

Figure 4 shows a pseudo-code of the GWO. The diversity of exploitation and exploration in the GWO was controlled by the vector $\vec{A}$. During the optimization process, when |A|< 1, the wolves tend to approach the prey because the next location of wolves is in the area between their current locations and the prey’s position^15^. This conforms the exploitation of the GWO. In contrast, as |A|> 1, wolves tend to diverge from the prey that confirms the global exploration. Besides, the vector $\vec{C}$ influences on the exploration and exploitation of the GWO because it is a random weight that affects the update of wolf location as shown in Eq. (5). This helps the GWO to overcome local optima.

### Wolf-inspired optimization and support vector regression for electricity use prediction.

**Figure 5:**
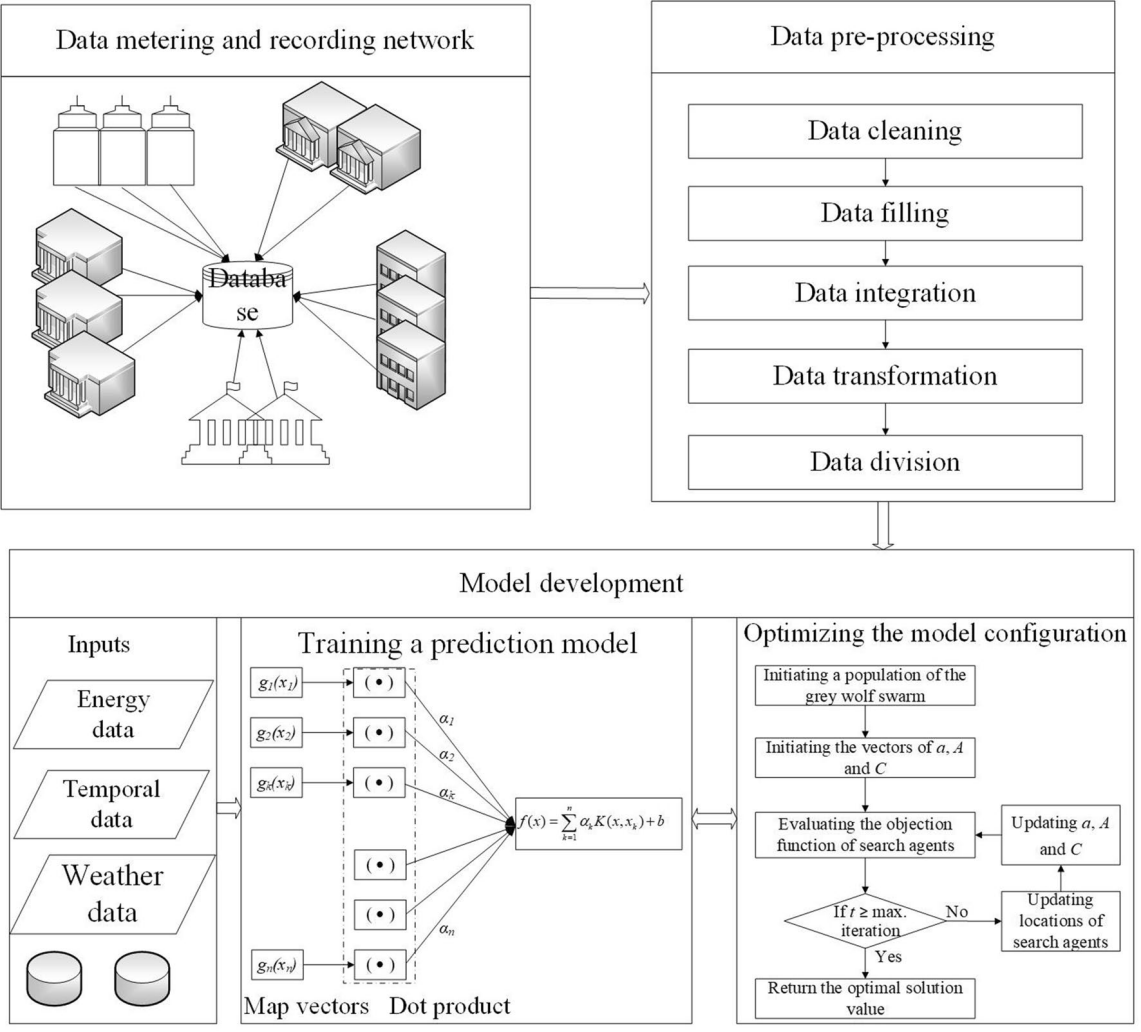
Implementation of wolf-inspired optimization and support vector regression.

Figure 5 presents the implementation of proposed WIO-SVR model that is an integration of the SVR model and the GWO algorithm. Energy data, weather data, and temporal data were collected. Particularly, energy consumption data in hundreds of buildings were collected using the data metering and recording network by the power company. Temporal data consists of day of the week and hour of the day that were extracted by the datetime in database.

These data were pre-processed before using for developing the prediction model. To improve performance of the model, the GWO optimized the model configuration by adjusting the SVR hyperparameters. The search space of the WIO-SVR model is set from 10^–3^ to 10^3^. The RBF was used as the kernel function in the SVR model. The population is 100 and the maximum iteration is 10. The optimization engine generated initially the population of the GWO in which their locations represent the *C* and *σ* values of the SVR in the search space. The optimal model is obtained as stopping criteria are reached. The proposed model was developed in the MATLAB environment which is a programming and numeric computing platform. The running environment is in the 64-bit operation system, the processor of Intel (R), core i7-8559U CPU @ 1.8 GHz 1.99 GHz, 8.00 GB RAM.

Criteria of evaluating the forecast performance were root-mean-square error (RMSE), mean absolute error (MAE), mean absolute percentage error (MAPE), and correlation coefficient (R). The RMSE measures the differences between predicted values and measured values. The MAE presents absolute errors between the predicted values and measured values. The MAPE shows accuracy in a percentage manner. The lower values of RMSE, MAE and MAPE indicate the better forecast accuracy. The R is used to measure the relationship between actual data and predicted data. The higher the absolute value of the R, the stronger the relationship. These measures are often used to assess the predictive performance of machine learning models^30^. Their corresponding equations are as follows.

11 $${\text{RMSE}} = \sqrt {\frac{1}{n}\sum\limits_{i = 1}^{n} {(y - y^{\prime})^{2} } } ,$$

12 $${\text{MAE}} = \frac{1}{n}\sum\limits_{i = 1}^{n} {\left| {y - y^{\prime}} \right|} ,$$

13 $${\text{MAPE}} = \frac{1}{n}\sum\limits_{i = 1}^{n} {\left| {\frac{{y - y^{\prime}}}{y}} \right|} ,$$

14 $${\text{R}} = \frac{{n\sum {y.y^{\prime} - (\sum y )(\sum {y^{\prime}} )} }}{{\sqrt {n(\sum {y^{2} } ) - (\sum y )^{2} } \sqrt {n(\sum {y^{{\prime}{2}} } ) - (\sum {y^{\prime}} )^{2} } }},$$

where *y* is the actual energy consumption, *y*′ is predicted energy consumption, and *n* is size of the data sample.

## Results

### Dataset

**Figure 6:**
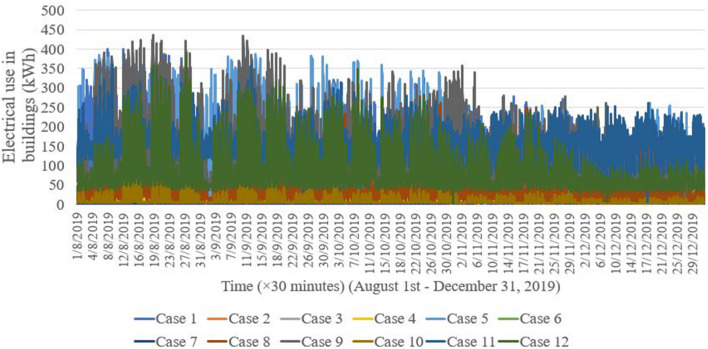
Electricity use profiles in 30-min resolution in 12 case studies.

**Figure 7:**
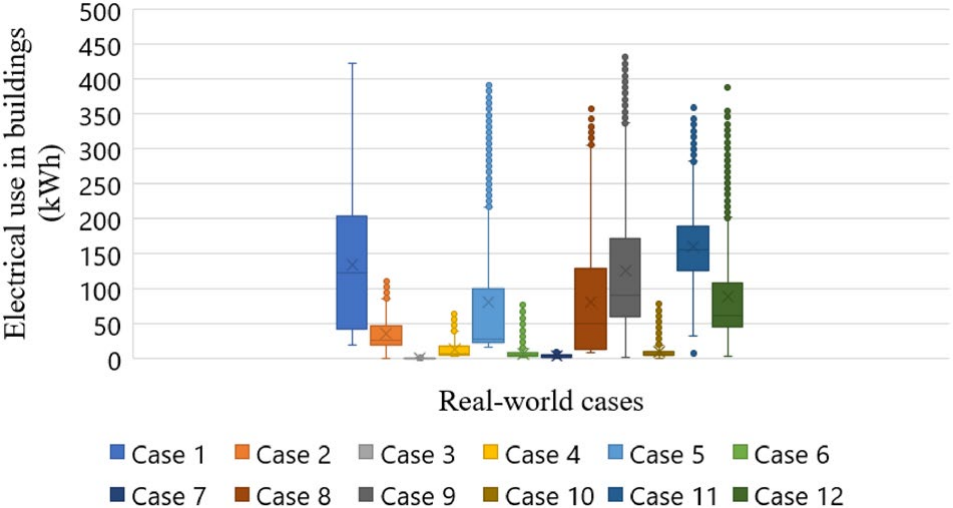
Box and Whisker plot of electrical use in 12 cases.

**Figure 8:**
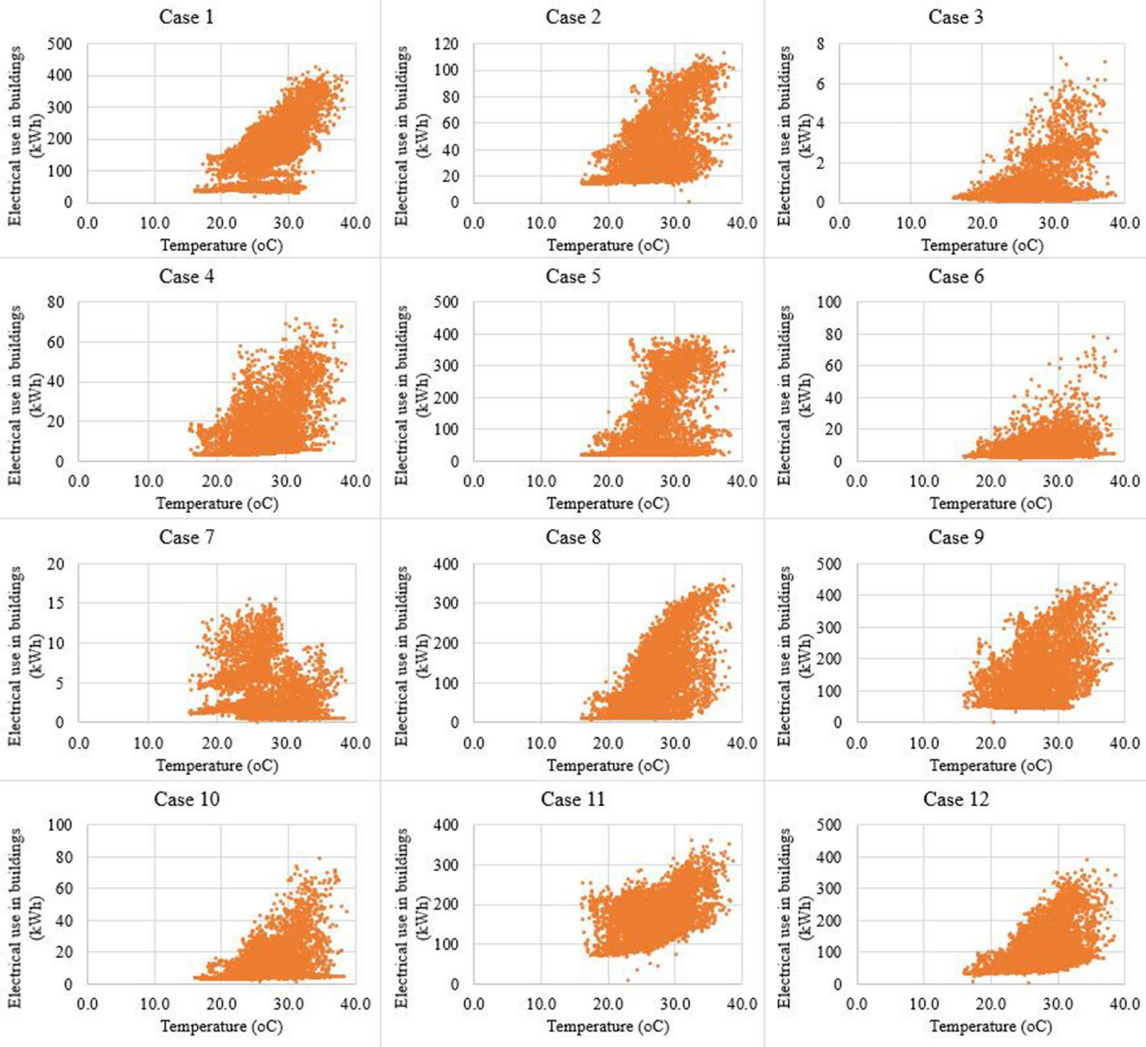
Scatter plots of energy data and temperature data.

Datasets in the 30-min interval in Danang city were collected to validate the proposed WIO-SVR model. Danang city was chosen as a case study in this study because Danang city is one of the biggest cities in Vietnam and Danang’s government has a policy to develop a smart city in near future. 12 buildings are used as case studies which include one commercial building, one hospital building, three authority buildings, three university buildings, and four office buildings (Table 1). The electrical usage patterns of 12 buildings are presented in Fig. 6. Figure 7 graphically depicts electrical consumption profiles using Box and Whisker method. Scatter plots of energy data and temperature data in Fig. 8 depict a positive correlation of energy and temperature data in all buildings. The WIO-SVR model aimed to predict 48-step-ahead energy consumption data which is a one-day-ahead prediction.

**Table 1:** Case studies.

Case	Building ID	Building type	Case	Building ID	Building type
1	41	Commercial building	7	419	Authority building
2	81	Office building	8	642	University building
3	87	Authority building	9	124	University building
4	79	Office building	10	136	Authority building
5	109	Office building	11	135	Hospital building
6	379	Office building	12	194	University building

The WIO-SVR model was evaluated multiple times using multiple datasets. Particularly, 12 datasets from 12 buildings were used and 14 evaluations were performed for each dataset. The original dataset is divided into learning data and test data. Learning data include 6856 observations in 4 months ranging from August 1st, 2019 to November 30th, 2019. Training data which accounted for 70% of learning data is used to train the SVR model. The rest of the learning data (i.e., validation data) are used to optimize the trained model. Test data that are utilized to test the prediction ability of the optimized WIO-SVR model include 672 observations in 14 days ranging from December 1st to 14th, 2019. Summation of model evaluation was as below:

- Prediction model: Wolf-inspired optimization support vector regression (WIO-SVR)
- Forecast period or model output: 1-day-ahead prediction or 48-step-ahead prediction (30-min interval data)
- Input: historical energy consumption data, outdoor temperature data, day of the week (i.e., Monday, Tuesday, Wednesday, Thursday, Friday, Saturday, Sunday) and hour of the day (i.e., 0, 1, 2, …, 21, 22, 23). These inputs have been recognized as model inputs for predicting energy consumption.
- Learning data: 4-month data, 01/8/2019–30/11/2019, size is 6856 data points.
- Test data: 01/12/2019–14/12/2019, size is 672 data points.

### Results and discussion

Learning data are divided into training data and validation data. Training data are used to train the WIO-SVR model while validation data are used to optimize the trained model. The forecast accuracy of the optimized WIO-SVR is evaluated by test data which ranges from December 1st to December 14th, 2019.

**Figure 9:**
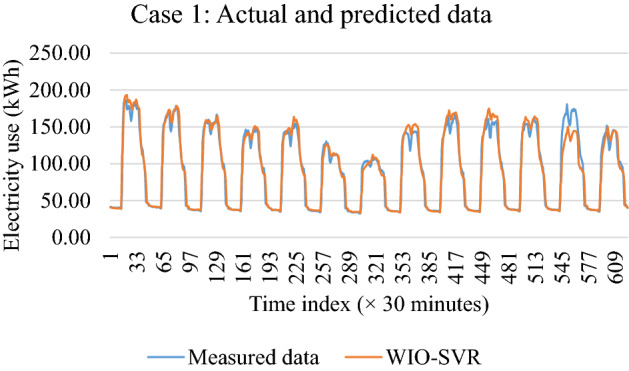
Actual and predicted data for case 1.

Table 2 presents performance measures of the proposed WIO-SVR model for case study 1 in terms of RMSE, MAE, MAPE, and R. The high values of R in both learning phase and test phase indicated a good agreement between observed and predicted data. The highest value of R in the learning phase and the test phase was 0.97 and 0.99, respectively. For the learning phase, the WIO-SVR obtained the best performance measures on December 14th with the RMSE value of 3.36 kWh. In the test phase, the proposed model presented a good prediction ability. The RMSE and MAE obtained smallest values on December 8th with 2.11 kWh and 4.44 kWh, respectively. Figure 9 displays actual and predicted values of electrical consumption in the test phase.

**Table 2:** Case study 1—evaluation results.

Test data for case 1	Learning WIO-SVR				Testing WIO-SVR			
	RMSE (kWh)	MAE (kWh)	MAPE (%)	R	RMSE (kWh)	MAE (kWh)	MAPE (%)	R
01/12/2019	3.46	11.95	10.17	0.97	2.78	7.75	8.53	0.97
02/12/2019	3.45	11.89	10.14	0.97	2.61	6.81	6.68	0.99
03/12/2019	3.44	11.83	10.09	0.97	2.41	5.83	6.53	0.98
04/12/2019	3.42	11.72	10.01	0.97	2.28	5.20	6.18	0.98
05/12/2019	3.40	11.53	9.86	0.97	2.32	5.38	6.36	0.98
06/12/2019	3.38	11.43	9.76	0.97	2.43	5.91	6.69	0.98
07/12/2019	3.37	11.34	9.69	0.97	2.15	4.63	6.64	0.97
08/12/2019	3.35	11.24	9.63	0.97	2.11	4.44	6.41	0.97
09/12/2019	3.33	11.10	9.59	0.97	2.63	6.93	7.34	0.98
10/12/2019	3.32	11.02	9.54	0.97	2.58	6.67	6.97	0.99
11/12/2019	3.31	10.95	9.51	0.97	2.81	7.88	7.76	0.98
12/12/2019	3.31	10.93	9.50	0.97	2.38	5.67	6.26	0.99
13/12/2019	3.29	10.84	9.46	0.97	2.87	8.23	8.10	0.99
14/12/2019	3.28	10.77	9.41	0.97	2.48	6.16	7.01	0.97
Average	3.36	11.32	9.74	0.97	2.49	6.25	6.96	0.98
Std. dev	0.06	0.41	0.27	0.00	0.24	1.18	0.72	0.01
Min	3.28	10.77	9.41	0.97	2.11	4.44	6.18	0.97
Max	3.46	11.95	10.17	0.97	2.87	8.23	8.53	0.99

**Figure 10:**
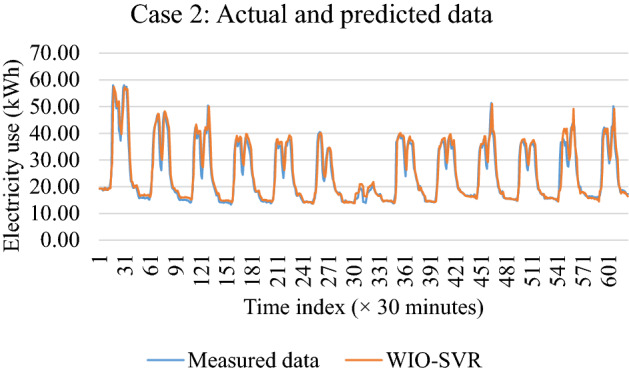
Actual and predicted data for case 2.

Table 3 shows the evaluation results of the WIO-SVR model for case study 2. This case study utilizes 30-min resolution data collected from the office building. The predictive accuracy of the proposed model in the test phase was much better than that in the learning phase. Three measures of RMSE, MAE, and MAPE in the test phase had smaller average values than those had in the learning phase. The proposed WIO-SVR model obtained an average of MAPE with 64.32% in the learning phase comparing to only 6.89% in the test phase. Figure 10 compares actual observations and predicted observations of the WIO-SVR using test data. Figure 10 reveals that predicted values well captured with actual values. Generally, the WIO-SVR model exhibited high forecast accuracy and the good agreement between actual data and predicted values.

**Table 3:** Case study 2—evaluation results.

Test data for case 2	Learning WIO-SVR				Testing WIO-SVR			
	RMSE (kWh)	MAE (kWh)	MAPE (%)	R	RMSE (kWh)	MAE (kWh)	MAPE (%)	R
01/12/2019	1.82	3.33	63.48	0.97	1.04	1.07	4.66	0.89
02/12/2019	1.82	3.30	63.45	0.97	1.64	2.68	7.37	0.95
03/12/2019	1.81	3.29	63.42	0.97	1.58	2.49	9.23	0.96
04/12/2019	1.81	3.28	63.49	0.97	1.63	2.66	10.06	0.95
05/12/2019	1.81	3.28	63.68	0.97	1.40	1.95	7.81	0.97
06/12/2019	1.80	3.25	64.08	0.97	1.33	1.77	6.92	0.96
07/12/2019	1.80	3.22	64.21	0.97	1.27	1.61	6.42	0.95
08/12/2019	1.79	3.20	64.29	0.97	1.01	1.01	6.20	0.87
09/12/2019	1.78	3.17	64.33	0.97	1.31	1.72	6.16	0.95
10/12/2019	1.78	3.18	64.74	0.96	1.22	1.50	5.51	0.97
11/12/2019	1.79	3.20	65.17	0.96	1.42	2.00	6.82	0.95
12/12/2019	1.79	3.22	65.50	0.96	1.21	1.46	5.55	0.96
13/12/2019	1.78	3.18	65.37	0.96	1.31	1.71	5.99	0.96
14/12/2019	1.77	3.15	65.26	0.96	1.51	2.29	7.78	0.94
Average	1.80	3.23	64.32	0.97	1.35	1.85	6.89	0.95
Std. dev	0.02	0.05	0.77	0.00	0.20	0.53	1.47	0.03
Min	1.77	3.15	63.42	0.96	1.01	1.01	4.66	0.87
Max	1.82	3.33	0.65	0.97	1.64	2.68	10.06	0.97

**Figure 11:**
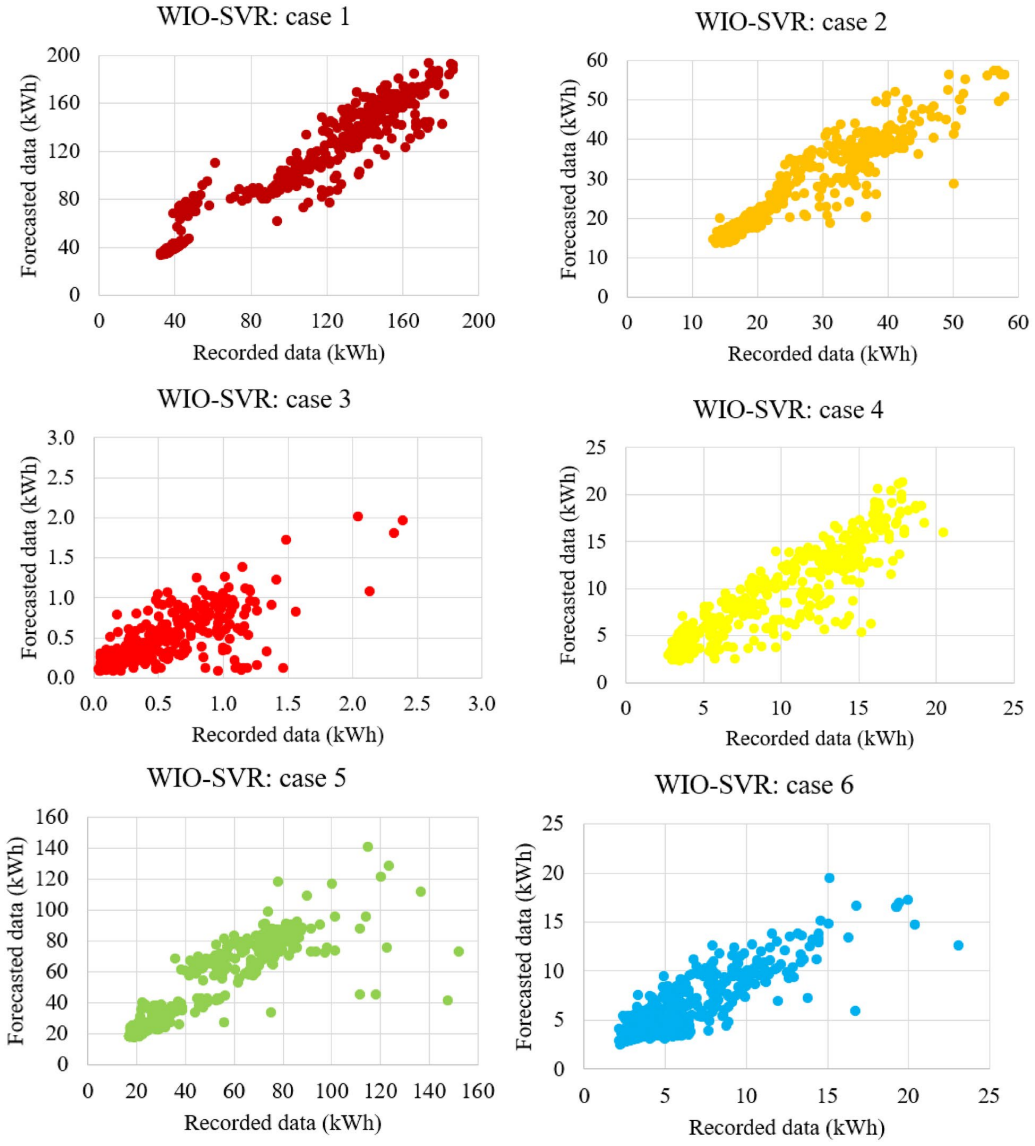

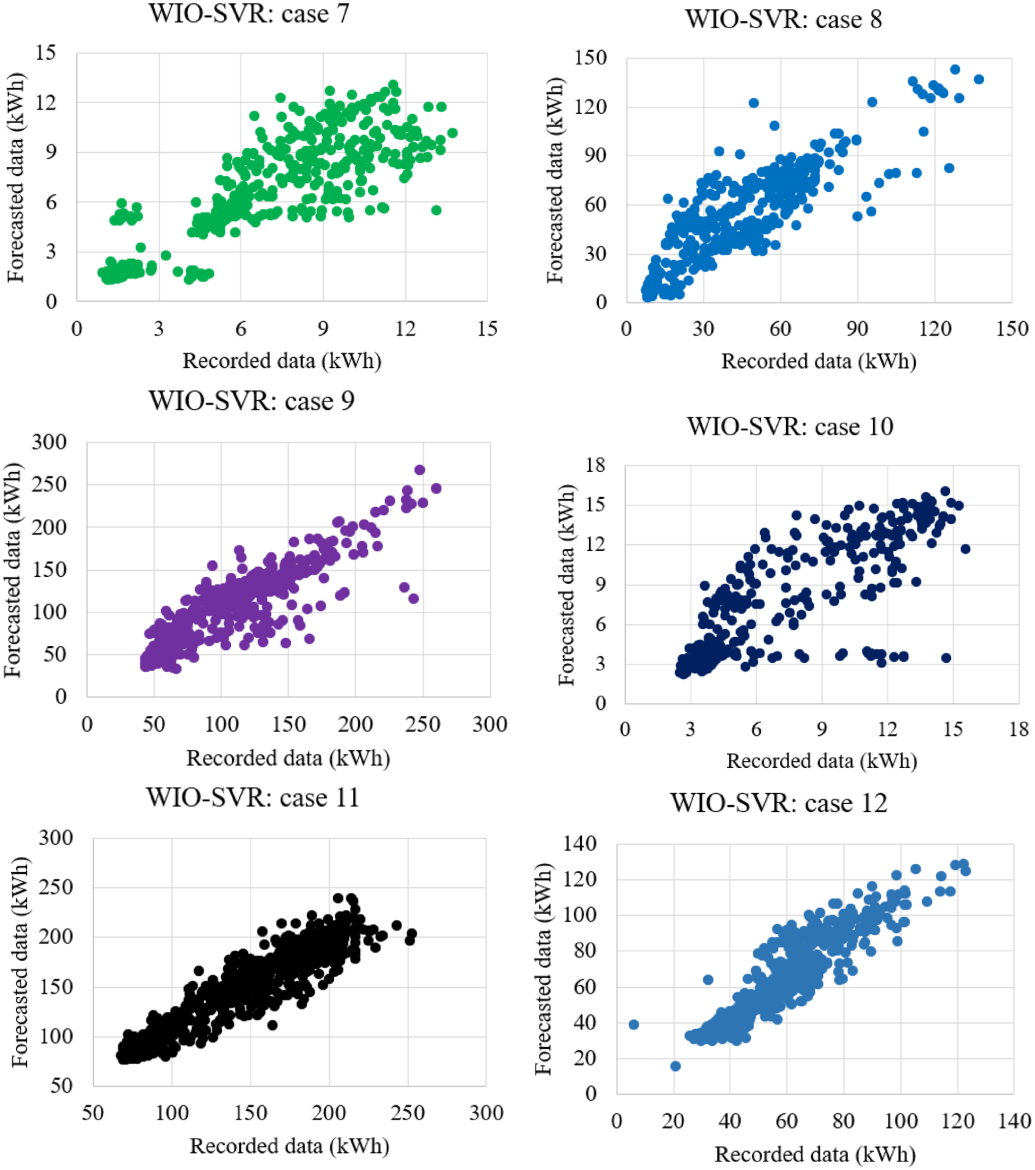
Scatter plots between recorded data and forecasted data by WIO-SVR in 12 cases.

Table 4 reported the predictive accuracy and standard deviation of the proposed WIO-SVR model in all cases. The smaller values of RMSE, MAE, and MAPE indicate the better predictive accuracy of the proposed model. The WIO-SVR yielded the smallest values of RMSE and MAE for the test data of the authority building in case 3. The values of RMSE and MAE were 0.31 kWh and 0.1 kWh, respectively. The smallest value of MAPE (6.96%) was obtained by the proposed model when using data collected from the commercial building (case 1). Among 12 cases, the WIO-SVR showed a lower predictive accuracy for case 11 with 3.63 kWh and 13.20 kWh in terms of RMSE and MAE, respectively. Particularly, the values of R in all cases are high which range from 0.78 to 0.98. This indicated that the proposed model is in good agreement with experimental datasets (Fig. 11).

**Table 4:** Accuracy of the WIO-SVR model for the test data.

Case	Building type	RMSE (kWh)		MAE (kWh)		MAPE (%)		R	
		Average	Std. dev	Average	Std. dev	Average	Std. dev	Average	Std. dev
1	Commercial building	2.49	0.24	6.25	1.18	6.96	0.72	0.98	0.01
2	Office building	1.35	0.20	1.85	0.53	6.89	1.47	0.95	0.03
3	Authority building	0.31	0.06	0.10	0.04	28.70	13.03	0.78	0.09
4	Office building	1.05	0.15	1.13	0.30	16.86	3.08	0.91	0.10
5	Office building	2.18	0.35	4.85	1.48	10.87	1.74	0.89	0.20
6	Office building	2.06	0.40	4.41	1.57	12.04	5.45	0.85	0.24
7	Authority building	1.00	0.11	1.00	0.19	20.40	2.50	0.90	0.03
8	University building	3.14	0.18	9.91	1.15	32.10	8.64	0.92	0.02
9	University building	3.52	0.25	12.44	1.81	13.26	1.59	0.91	0.02
10	Authority building	0.90	0.29	0.89	0.45	15.18	6.03	0.83	0.14
11	Hospital building	3.63	0.18	13.20	1.33	9.77	0.95	0.92	0.02
12	University building	2.63	0.35	7.01	1.81	12.88	2.91	0.92	0.06

To confirm the effectiveness of the proposed WIO-SVR model, its predictive accuracy was compared with those of machine learning models including the SVR, random forest (RF), M5P, and REPTree. These models are popular machine learning models and they were often used in building energy consumption prediction. Table 5 indicates that the predictive ability of the WIO-SVR was superior to that of baseline models in all cases. The R values of the proposed model in all cases were high indicating a good agreement between real data and predicted data. For the dataset collected from the commercial building (case 1), all performance measures of the WIO-SVR outperformed those of SVR, RF, M5P, and REPTree. For example, the WIO-SVR yielded the smallest values of RMSE with 2.49 kWh, followed by 9.52 kWh and 21.02 kWh of SVR and RF, respectively.

**Table 5:** Performance comparison among prediction models.

Case	Accuracy	WIO-SVR	SVR	RF	M5P	REPTree
Case 1	RMSE (kWh)	2.49	9.52	21.02	33.51	52.35
	MAE (kWh)	6.25	7.36	14.83	23.76	37.35
	MAPE (%)	6.96	9.99	14.46	24.19	33.21
	R	0.98	0.99	0.97	0.76	0.91
Case 2	RMSE (kWh)	1.35	5.57	8.36	11.05	28.43
	MAE (kWh)	1.85	3.98	6.50	8.49	8.60
	MAPE (%)	6.89	16.57	25.78	33.19	12.46
	R	0.95	0.88	0.91	0.62	0.38
Case 3	RMSE (kWh)	0.31	0.31	0.36	0.46	1.21
	MAE (kWh)	0.10	0.19	0.25	0.36	0.70
	MAPE (%)	28.70	57.98	143.96	127.82	304.43
	R	0.78	0.56	0.40	0.36	0.16
Case 4	RMSE (kWh)	1.05	2.08	3.10	3.51	6.72
	MAE (kWh)	1.13	1.47	2.04	2.58	4.23
	MAPE (%)	16.86	21.83	25.75	40.25	46.63
	R	0.91	0.90	0.92	0.77	0.88
Case 5	RMSE (kWh)	2.18	18.85	50.47	47.57	31.32
	MAE (kWh)	4.85	12.02	31.77	27.18	20.31
	MAPE (%)	10.87	24.83	67.22	58.59	49.00
	R	0.89	0.84	0.84	0.67	0.78
Case 6	RMSE (kWh)	2.06	2.42	4.65	4.18	5.89
	MAE (kWh)	4.41	1.65	3.01	2.59	3.96
	MAPE (%)	12.04	28.28	59.01	48.95	83.55
	R	0.85	0.74	0.66	0.62	0.59
Case 7	RMSE (kWh)	1.00	1.59	1.47	1.77	3.63
	MAE (kWh)	1.00	1.28	1.06	1.35	2.98
	MAPE (%)	20.40	40.10	25.42	39.90	143.88
	R	0.90	0.91	0.92	0.89	0.55
Case 8	RMSE (kWh)	3.14	24.99	35.52	24.48	47.49
	MAE (kWh)	9.91	17.53	25.89	17.21	33.82
	MAPE (%)	32.10	48.01	67.35	51.23	80.77
	R	0.92	0.94	0.92	0.87	0.92
Case 9	RMSE (kWh)	3.52	28.87	28.24	35.95	43.18
	MAE (kWh)	12.44	17.66	18.15	24.32	27.99
	MAPE (%)	13.26	17.68	18.84	25.37	35.26
	R	0.91	0.80	0.84	0.68	0.71
Case 10	RMSE (kWh)	0.90	1.48	5.53	3.83	7.03
	MAE (kWh)	0.89	0.95	3.91	2.65	4.42
	MAPE (%)	15.18	18.43	71.49	43.76	62.80
	R	0.83	0.91	0.88	0.88	0.94
Case 11	RMSE (kWh)	3.63	19.36	17.43	21.78	36.77
	MAE (kWh)	13.20	14.38	14.40	17.13	30.68
	MAPE (%)	9.77	9.55	11.89	12.88	27.07
	R	0.92	0.92	0.95	0.88	0.68
Case 12	RMSE (kWh)	2.63	16.42	19.12	24.73	53.22
	MAE (kWh)	7.01	12.13	14.12	19.89	38.21
	MAPE (%)	12.88	21.04	25.22	37.36	64.93
	R	0.92	0.91	0.91	0.88	0.80

With regard to the dataset collected from office buildings (cases 2, 4, 5, and 6), the proposed model showed its outperformance in predicting electrical consumption. Notably, the RMSE and the MAE of the WIO-SVR model in case 5 were only 2.18 kWh and 4.85 kWh, respectively. These measures were significantly lower than those of other models. In case 6, although the WIO-SVR had a higher value of MAE than other models, the RMSE, MAE, and MAPE of this model were much lower than baseline models.

Similarly, the evaluation results of the WIO-SVR model for datasets collected from educational buildings (cases 8, 9, and 12) were significantly better than those of other ML models. Table 5 showed that the RMSE obtained by the proposed model in case 8 and case 9 was 3.14 kWh and 3.52 kWh, respectively. These measures were lowest among RMSE’s values obtained by SVR, RF, M5P, and REPTree. Notably, all models in case 8 exhibited higher MAPE values than in the remaining cases. With the hospital building, the proposed model confirmed its capacity in forecasting electricity usage.

**Figure 12:**
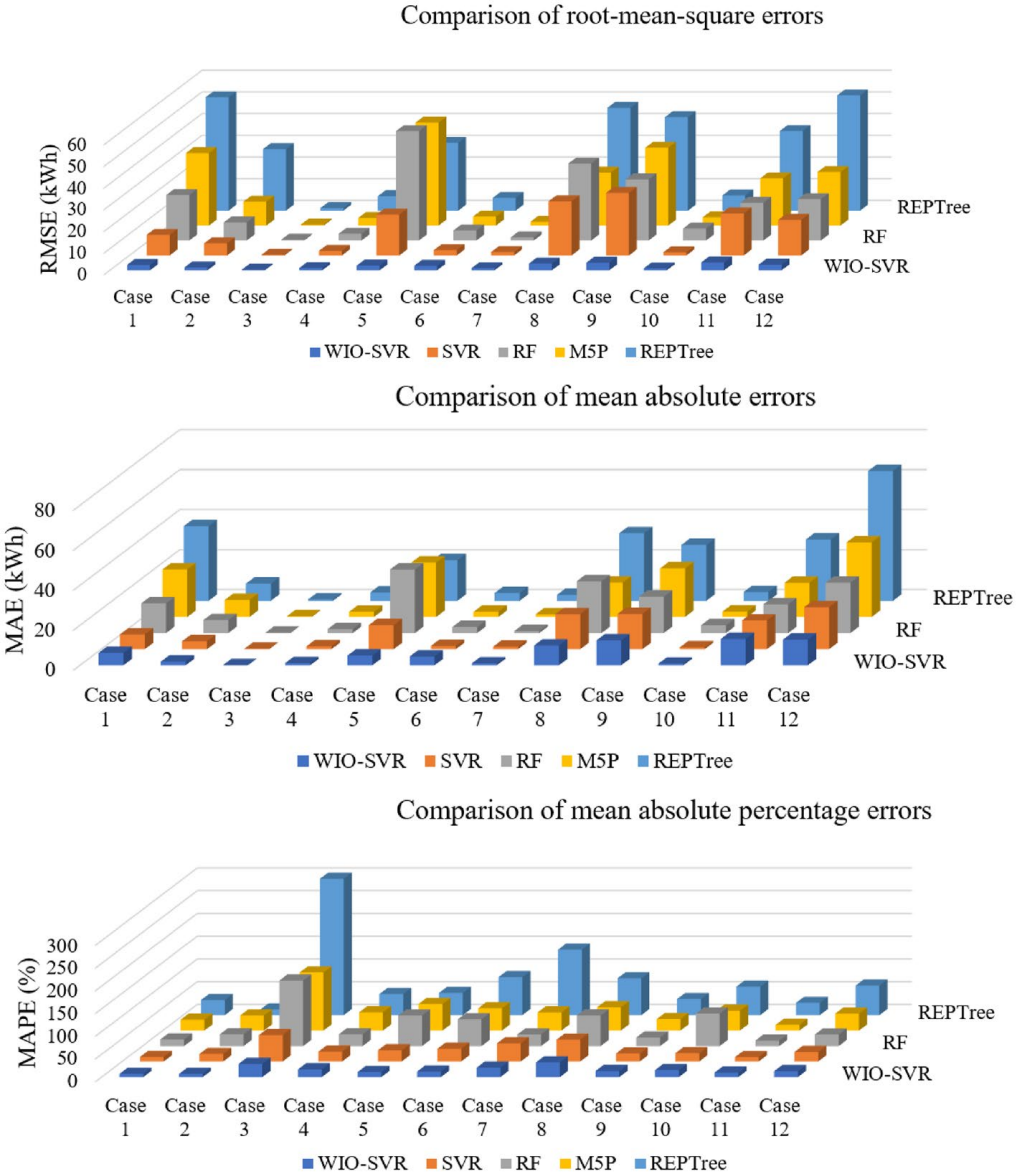
Performance comparison of all models.

**Figure 13:**
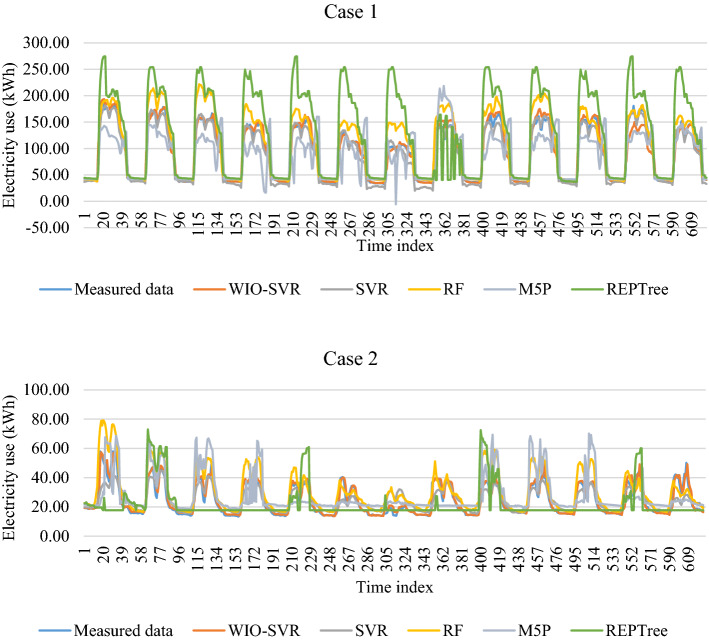
The actual values and predicted values obtained by all models for cases 1 and 2.

For authority buildings (cases 3, 7, and 10), the proposed model still maintained lower prediction errors than comparative models. In particular, the MAPE obtained by the five models was higher than that obtained by them in cases where data collected from commercial, hospital, and office buildings. Figure 12 compares measures of RMSE, MAE, and MAPE obtained by the proposed model and other ML models. The actual values and predicted values obtained by all models for case 1 and case 2 were presented in Fig. 13. The predicted values yielded by the WIO-SVR model were well captured with the actual ones.

The outstanding efficiency of the proposed model was confirmed in Table 6. Among the models, the proposed WIO-SVR model obtained the highest value of R with 0.90. The average value of the RMSE of the WIO-SVR was 2.02 kWh which was much lower than that of the SVR with 10.95 kWh, the RF with 16.27 kWh, the M5P with 17.73 kWh, and the REPTree with 26.44 kWh. The proposed model improved 442.0–1207.9% and 43.7–238.3% the predictive accuracy in terms of the RMSE and the MAE, respectively. Overall, the error rates of the WIO-SVR model are 43.7–1027.9% better than those of baseline models. Thus, the proposed WIO-SVR model was suggested as an effective model for forecasting energy consumption in non-residential buildings.

**Table 6:** Outstanding performance by the WIO-SVR model.

Statistical indices		Intelligent prediction model					Accuracy change by WIO-SVR model			
		WIO-SVR	SVR	RF	M5P	REPTree	SVR	RF	M5P	REPTree
RMSE (kWh)	Average	2.02	10.95	16.27	17.73	26.44	442.0^a^	705.0^a^	777.4^a^	1207.9^a^
	Std. dev	1.10	10.24	15.59	15.84	20.43				
MAE (kWh)	Average	5.25	7.55	11.33	12.29	17.77	43.7^c^	115.6^a^	134.0^a^	238.2^a^
	Std. dev	4.61	6.82	10.30	10.27	15.02				
MAPE (%)	Average	15.5	26.2	46.4	45.3	78.7	69.1^a^	199.3^a^	192.4^a^	407.8^a^
	Std. dev	8.0	15.1	38.0	28.9	79.0				
R	Average	0.90	0.86	0.84	0.74	0.69	4.3^b^	6.0^a^	17.4^a^	22.9^a^
	Std. dev	0.05	0.12	0.16	0.16	0.24				

^a^Indicates the significant level less than 1%.

^b^Indicates the significant level less than 5%.

^c^Indicates the significant level less than 10%.

This study proposed the hybrid machine learning model that integrates the GWO and SVR model to enhance the predictive performance of energy use prediction in non-residential buildings. Evaluation results revealed that the performance of the proposed WIO-SVR model was better than those of investigated machine learning model. This is because the hyperparameters of the SVR model were optimized by an optimization algorithm (i.e., GWO) during the learning process. Therefore, the WIO-SVR can be used as a tool for time-series energy consumption prediction in non-residential buildings.

## Conclusions

The building sector has considered the largest consumer of energy that accounts for nearly 40% of global energy usage and 33% of greenhouse gas emissions. Electricity demand forecasting is an essential task since it supports making important decisions in power system planning and operation. An accurate energy demand forecast model supports the decision-makers to carry out short-term management of the electric utility. Meanwhile, machine learning models have become a mainstream of modeling building energy. This study develops a hybrid model of wolf-inspired optimized support vector regression (WIO-SVR) for energy demand forecasting in non-residential buildings.

Electrical consumption data in 30-min interval collected in Danang city, the largest city in the central land of Vietnam, were adopted to validate the performance of the WIO-SVR. Twelve buildings are used as case studies which include one commercial building, one hospital building, three authority buildings, three university buildings, and four office buildings. The WIO-SVR model aimed to predict 48-step-ahead energy consumption data which is a one-day-ahead prediction. The performance of the proposed model is compared with those of benchmark models.

The original dataset is divided into learning data and test data. Learning data include 6856 observations in 4 months ranging from August 1st, 2019 to November 30th, 2019. Training data which accounted for 70% of learning data is used to train the SVR model. The rest of the learning data (i.e., validation data) are used to optimize the trained model. Test data that are utilized to test the prediction ability of the optimized WIO-SVR model include 672 observations in 14 days ranging from December 1st to 14th, 2019. The WIO-SVR model was evaluated multiple times using multiple datasets. Particularly, 12 datasets from 12 buildings were used and 14 evaluations were performed for each dataset.

Root-mean-square error (RMSE), mean absolute error (MAE), mean absolute percentage error (MAPE), and correlation coefficient (R) were adopted as evaluation criteria of model performance. The smaller values of RMSE, MAE, and MAPE indicate the better predictive accuracy of prediction models. The WIO-SVR yielded the smallest values of RMSE and MAE for the test data of the authority building in case 3. The values of RMSE and MAE were 0.31 kWh and 0.1 kWh, respectively. The smallest value of MAPE (6.96%) was obtained by the proposed model when using data collected from the commercial building (case 1). Among 12 cases, the WIO-SVR showed a lower predictive accuracy for case 11 with 3.63 kWh and 13.20 kWh in terms of RMSE and MAE, respectively. Particularly, the values of R in all cases are high which range from 0.78 to 0.98. This indicated that the proposed model is in good agreement with experimental datasets.

To confirm the effectiveness of the proposed WIO-SVR model, its predictive accuracy was compared with those of machine learning models including the SVR, random forest (RF), M5P, and REPTree. The predictive ability of the WIO-SVR was superior to that of baseline models in all cases. The R values of the proposed model in all cases were high indicating a good agreement between real data and predicted data. For the dataset collected from the commercial building (case 1), all performance measures of the WIO-SVR outperformed those of SVR, RF, M5P, and REPTree. For example, the WIO-SVR yielded the smallest values of RMSE with 2.49 kWh, followed by 9.52 kWh and 21.02 kWh of SVR and RF, respectively.

Among the models, the proposed WIO-SVR model obtained the highest value of R with 0.90. The average value of the RMSE of the WIO-SVR was 2.02 kWh which was much lower than that of the SVR with 10.95 kWh, the RF with 16.27 kWh, the M5P with 17.73 kWh, and the REPTree with 26.44 kWh. The proposed model improved 442.0–1207.9% and 43.7–238.3% the predictive accuracy in terms of the RMSE and the MAE, respectively. Overall, the error rates of the WIO-SVR model are 43.7–1027.9% better than those of baseline models. As the study`s contribution, an effective and reliable WIO-SVR model provides building managers with useful references in proactively efficient energy management.

This study contributes to (i) the state of the knowledge by examining the performance of WIO-SVR model in predicting time-series energy data; and (ii) the state of practice by proposing an effective tool to help building owners and facility managers in understanding building energy performance for enhancing the energy efficiency in buildings.

As a limitation, the proposed method requires users with basic programming skills and machine learning knowledge. In this study, the GWO was used as an optimization engine in the proposed model. Future studies may investigate other optimization algorithms such as monarch butterfly optimization, earthworm optimization algorithm, elephant herding optimization, moth search algorithm, Slime mould algorithm, and Harris hawks optimization to improve performance of the prediction model.

## References

[1] Ngo N-T, Truong TTH (2019). Forecasting time series data using moving-window swarm intelligence-optimised machine learning regression. Int. J. Intell. Eng. Inform..

[2] Spandagos C, Ng TL (2017). Equivalent full-load hours for assessing climate change impact on building cooling and heating energy consumption in large Asian cities. Appl. Energy.

[3] Liu T, Tan Z, Xu C, Chen H, Li Z (2020). Study on deep reinforcement learning techniques for building energy consumption forecasting. Energy Build..

[4] Injadat M, Moubayed A, Nassif AB, Shami A (2021). Machine learning towards intelligent systems: Applications, challenges, and opportunities. Artif. Intell. Rev..

[5] Zhu J, Shen Y, Song Z, Zhou D, Zhang Z, Kusiak A (2019). Data-driven building load profiling and energy management. Sustain. Cities Soc..

[6] Xue X (2017). Prediction of slope stability based on hybrid PSO and LSSVM. J. Comput. Civ. Eng..

[7] Bourdeau M, Zhai X, Nefzaoui E, Guo X, Chatellier P (2019). Modeling and forecasting building energy consumption: A review of data-driven techniques. Sustain. Cities Soc..

[8] Suykens JAK, Gestel TV, Brabanter JD, Moor BD, Vandewalle J (2002). Least Squares Support Vector Machines.

[9] Breiman LJML (2001). Random Forests. Machine Learning.

[10] Yi J-H, Wang J, Wang G-G (2016). Improved probabilistic neural networks with self-adaptive strategies for transformer fault diagnosis problem. Adv. Mech. Eng..

[11] Wang G-G, Lu M, Dong Y-Q, Zhao X-J (2016). Self-adaptive extreme learning machine. Neural Comput. Appl..

[12] Pan Y, Zhang L (2020). Data-driven estimation of building energy consumption with multi-source heterogeneous data. Appl. Energy..

[13] Pham A-D, Ngo N-T, Ha Truong TT, Huynh N-T, Truong N-S (2020). Predicting energy consumption in multiple buildings using machine learning for improving energy efficiency and sustainability. J. Clean. Prod..

[14] Chen Y, Zhang F, Berardi U (2020). Day-ahead prediction of hourly subentry energy consumption in the building sector using pattern recognition algorithms. Energy.

[15] Mirjalili S, Mirjalili SM, Lewis A (2014). Grey wolf optimizer. Adv. Eng. Softw..

[16] Goudarzi S, Anisi MH, Kama N, Doctor F, Soleymani SA, Sangaiah AK (2019). Predictive modelling of building energy consumption based on a hybrid nature-inspired optimization algorithm. Energy Build..

[17] Somu N, Gauthama Raman MR, Ramamritham K (2020). A hybrid model for building energy consumption forecasting using long short term memory networks. Appl. Energy..

[18] González-Vidal A, Jiménez F, Gómez-Skarmeta AF (2019). A methodology for energy multivariate time series forecasting in smart buildings based on feature selection. Energy Build..

[19] Choubey S, Karmakar GP (2021). Artificial intelligence techniques and their application in oil and gas industry. Artif. Intell. Rev..

[20] Banan A, Nasiri A, Taheri-Garavand A (2020). Deep learning-based appearance features extraction for automated carp species identification. Aquacult. Eng..

[21] Wu CL, Chau KW (2013). Prediction of rainfall time series using modular soft computingmethods. Eng. Appl. Artif. Intell..

[22] Mostashari-Rad F, Nabavi-Pelesaraei A, Soheilifard F, Hosseini-Fashami F, Chau K-W (2019). Energy optimization and greenhouse gas emissions mitigation for agricultural and horticultural systems in Northern Iran. Energy.

[23] Biswas MAR, Robinson MD, Fumo N (2016). Prediction of residential building energy consumption: A neural network approach. Energy.

[24] Lu H, Cheng F, Ma X, Hu G (2020). Short-term prediction of building energy consumption employing an improved extreme gradient boosting model: A case study of an intake tower. Energy.

[25] Ma S (2021). A hybrid deep meta-ensemble networks with application in electric utility industry load forecasting. Inf. Sci..

[26] Ferreira TM, Estêvão J, Maio R, Vicente R (2020). The use of Artificial Neural Networks to estimate seismic damage and derive vulnerability functions for traditional masonry. Front. Struct. Civ. Eng..

[27] Tian Y, Yu J, Zhao A (2020). Predictive model of energy consumption for office building by using improved GWO-BP. Energy Rep..

[28] Chou J-S, Truong TTH (2019). Sliding-window metaheuristic optimization-based forecast system for foreign exchange analysis. Soft. Comput..

[29] Chou J-S, Ngo N-T (2018). Engineering strength of fiber-reinforced soil estimated by swarm intelligence optimized regression system. Neural Comput. Appl..

[30] Zeinolabedini M, Najafzadeh M (2019). Comparative study of different wavelet-based neural network models to predict sewage sludge quantity in wastewater treatment plant. Environ. Monit. Assess..

